# TIO Associated with Hyperparathyroidism: A Rarity, a Rule, or a Novel HPT-PMT Syndrome—A Case Study with Literature Review

**DOI:** 10.1155/2021/5172131

**Published:** 2021-07-26

**Authors:** Michael Salim, Mohannad Samy Behairy, Elena Barengolts

**Affiliations:** ^1^Department of Internal Medicine, Mount Sinai Hospital, Chicago, IL 60608, USA; ^2^Ross University School of Medicine, Bridgetown, Barbados; ^3^Department of Medicine, University of Illinois Medical Center and Department of Medicine, Jesse Brown VA Medical Center, Chicago, IL 60612, USA

## Abstract

**Objective:**

Association of primary hyperparathyroidism (pHPT) with phosphaturic mesenchymal tumors (PMT) is rarely reported. This report entertains the hypothesis of the causal association of HPT with tumor-induced osteomalacia (TIO) and of the existence of HPT-PMT syndrome. *Case Presentation*. A 49-year-old man presented with fragility rib fractures, generalized bone pain, and muscle weakness worsening over the past 3 years. Initial tests demonstrated hypophosphatemia and high PTH. The diagnosis of pHPT was entertained, but parathyroid scan was negative. During a 2-year follow-up, the patient reported minimal improvement of symptoms after intermittent treatment with calcitriol and phosphate. Biochemical evaluation showed persistent hypophosphatemia with renal phosphate wasting, elevated FGF23, and osteopenia on DXA scan. TIO was suspected. Multiple MRIs and whole-body FDG-PET scans were inconclusive. The patient subsequently underwent ^68^Ga-DOTATATE PET-CT, which revealed a somatostatin receptor-positive lesion in the lung. The resected mass was confirmed as PMT. The patient had dramatically improved symptoms, normal phosphate, calcium, and FGF23. During follow-up over 3 years postsurgery, the patient had slowly rising calcium and persistently elevated PTH.

**Conclusion:**

The debate whether the patient had pHPT or tertiary HPT prompted literature review showing that aberrant genes including FGFR1, FGF1, fibronectin 1, and Klotho were mechanistically involved in the HPT-PMT association. This case highlights the pitfalls contributing to delayed diagnosis and treatment of TIO and hypothesizes the association between pHPT and PMT.

## 1. Introduction

Tumor-induced osteomalacia (TIO) is a rare paraneoplastic syndrome caused by phosphaturic mesenchymal tumors (PMT) secreting fibroblast growth factor 23 (FGF23) [1]. Physiologically produced predominantly by osteocytes and osteogenic cells, FGF23, a peptide hormone, is the primary regulator of serum phosphate (phosphatonin) through its effects on the parathyroid glands, kidneys, and vitamin D metabolism. Clinically, TIO presents with myalgia, bone pain, osteomalacia, and fragility fractures; the laboratory hallmarks of TIO are renal phosphate wasting, hypophosphatemia, low or inappropriately normal vitamin D, and normal serum calcium and parathyroid hormone (PTH) [1]. Definitive treatment of TIO is surgical resection of the offending tumor followed by biochemical and clinical resolution of the disease state. The rarity of the disorder, difficulty in tumor localization, and potential comorbidities often make TIO diagnosis and treatment challenging and delayed [1]. Increased or inappropriately normal (for the degree of hypophosphatemia) PTH is rarely diagnosed as hyperparathyroidism (HPT) [2]. We report a case of a patient with PMT and primary hyperparathyroidism (pHPT) and possible existence of HPT-PMT syndrome.

## 2. Case Presentation

A 49-year-old man was seen in the endocrinology clinic with a 3-year history of fragility rib fractures, muscle weakness, and generalized bone pain unrelieved with high-dose opioids. Rheumatologic workup was negative. Initial tests showed elevated circulating PTH 114.1 pg/mL (14–72 pg/mL) and alkaline phosphatase (ALP) 283 IU/L (44–174 IU/L), reduced phosphate 1.6 mg/dL (2.5–4.9 mg/dL), 25(OH)D 16 ng/mL (30–100 ng/mL), 1,25(OH)_2_D 9 pg/mL (18–72 pg/mL), and eGFR 58 mL/min/1.73 m^2^, and normal calcium level 9.2 mg/dL (8.5–10.1 mg/dL) (Table 1). The diagnosis of primary normocalcemic HPT or secondary HPT (sHPT, due to vitamin D deficiency) was entertained. A sestamibi scan showed normal parathyroid uptake.

**Figure 1:**
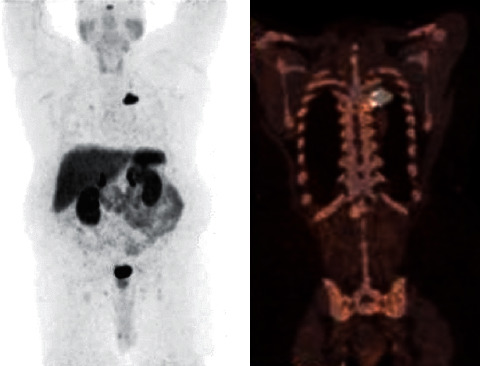
^68^Ga-DOTATATE PET-CT maximum intensity projection coronal (a) and representative-fused PET/CT coronal (b) views demonstrating somatostatin receptor-positive lesion involving the posteromedially located pleural-based 3.5 cm mass in the left upper lobe.

During a 2-year follow-up, the patient was intermittently taking vitamin D, calcitriol, and phosphate (phosphate in low dose and with poor adherence due to side effects) and reported some improvement of symptoms. Further investigations showed persistent hypophosphatemia with renal phosphate wasting (elevated urinary fractional phosphate excretion 44%, ref. <20%), high FGF23 291 RU/mL (0–180 RU/mL), and osteopenia on DXA. TIO due to PMT (TIO-PMT) was suspected. Over the next few years, investigations included three whole-body FDG PET-CT scans revealing several suspicious areas, yet no tumor was seen on multiple MRIs. Follow-up laboratory tests showed persistent hypophosphatemia, increasing FGF23 (up to 1330 RU/mL) and PTH (up to 274.4 pg/mL), and normal calcium, 25(OH)D, and 1,25(OH)_2_D (Table 1). The ^68^Ga-DOTATATE PET-CT revealed a somatostatin receptor-positive lesion involving the left upper lobe of the lung (Figure 1).

**Figure 2:**
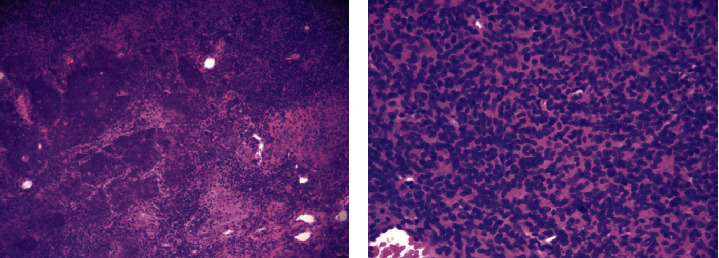
Histopathologic images showing hemangiopericytoma-like vasculature and calcified chondroid-like matrix in intermediate power (a) and cellular spindle cell lesion in high power (b) consistent with mesenchymal tumor.

The lung mass was resected with histopathology confirming PMT (Figure 2). At a 6-month postoperative follow-up, the patient reported dramatically improved symptoms, and tests showed normal phosphate, calcium, ALP, and FGF23 (160 RU/mL) (Table 1) and improved DXA. Phosphorus supplementation was discontinued. At follow-up 3 years postsurgery, the patient was asymptomatic, and tests showed normal phosphate, 25(OH)D, and FGF23 (174 RU/mL) but high calcium (10.6 mg/dL) and PTH (126.3 pg/mL) (Table 1). A diagnosis of tertiary HPT (tHPT) was suspected, but the patient did not meet the criteria for surgical treatment, so no investigations were done. The nature of HPT, whether pHPT (since PTH was high at the initial presentation) or tHPT (due to long-standing hypovitaminosis D), was debated and prompted literature review.

## 3. Discussion

The patient was initially diagnosed as pHPT with a normal parathyroid scan, similar to previous reports in TIO-PMT [3]. Hyperparathyroidism was uncommonly acknowledged in TIO-PMT cases [1, 2], and predominantly, HPT was diagnosed as sHPT [1] or tHPT [1, 4]. Tertiary HPT would not apply to our case since PTH was high at the initial presentation. Secondary HPT was described in TIO-PMT and explained by the suppressive action of FGF23 on renal 25(OH)D-1-*α*-hydroxylase [5], similar to FGF23-induced TIO in animal models, showing HPT and parathyroid hyperplasia [6]. Vitamin D and specifically calcitriol usually normalized PTH in sHPT [5]. In our patient, two years of vitamin D and calcitriol treatment normalized 25(OH)D and 1,25(OH)_2_D, but PTH remained high, suggesting factors other than or in addition to vitamin D deficiency contributed to HPT. Primary HPT was rarely diagnosed in patients presenting with TIO. In our case, there is the possibility that the patient had primary normocalcemic hyperparathyroidism at the beginning evolving into primary hypercalcemic hyperparathyroidism. In a published case diagnosed as pHPT and TIO due to unlocalizable PMT, the patient was treated with total (four glands) parathyroidectomy [3]. Particularly, insightful was a case of a young patient with a de novo germline translocation involving *α*-Klotho gene resulting in high circulating *α*-Klotho, FGF23, and PTH [7]. The patient was diagnosed and treated for hypophosphatemic rickets. There was no localizable PMT, yet, 30 years later, the patient developed two PMT in the mastoid bone [8]. The published reports and our case could imply that the association of PMT and HPT was not accidental, and perhaps, FGF23 per se or FGF23 pathway (including FGFR1-Klotho receptor) was the primary inducer of hyperparathyroidism.

Accumulating research has provided new insights showing that FGF23 is an important ligand for the FGFR1-Klotho receptor pathway playing a pivotal role in FGF23 hypersecretion phenotype and pathogenesis of PMT [8, 9]. FGFR1, a cell surface receptor with tyrosine kinase activity, works in union with its obligatory coreceptor Klotho, a transmembrane protein with a *ß*-glucuronidase activity. Both FGF23 and Klotho can be cleaved and shed and act as circulating hormones. The binding of FGF23 to FGFR1-Klotho activates downstream events regulating mineral metabolism, cell differentiation, and aging [8, 9]. Specifically, fusion of fibronectin 1 (FN1) with FGFR1 or FGF1 creates aberrant fusion genes FN1-FGFR1 and FN1-FGF1, which are overexpressed in 42% and 6% of PMT, respectively [9], while aberrant *α*-Klotho (or less commonly *β*-Klotho) genes are overexpressed in 8 of 10 fusion-negative PMT [8]. These aberrant genes activate the FGF1-FGFR signaling pathway resulting in paracrine positive feedback with upregulation of FGF23 gene and uncontrolled secretion of FGF23 by PMT [8, 9].

There are no published studies exploring mechanisms of the association between PMT and HPT. In clinical cases, pHPT has been reported as rare and accidental [3]. Alternatively, however, the coexistence of HPT and PMT may not be accidental but related to common factors contributing to the development of HPT-PMT syndrome similar to HPT-jaw tumor syndrome [10]. Review of literature reveals that several genes can be implicated as potential candidates for inducing PMT and HPT. Aberrant genes overexpressed in PMT can also initiate changes in parathyroid glands resulting in hyperplasia and autonomous secretion of PTH. Consistent with this hypothesis, published data show that parathyroid glands had an abundant expression of FGF1-*α*-Klotho receptors [10, 11]. Correspondingly, in clinical HPT due to parathyroid tumors, frequent upregulation of FGF1 and FGFR1 genes has been reported [10]. In experimental models of HPT, FGF23–FGFR1-Klotho signaling upregulates PTH gene expression, PTH secretion, and parathyroid cell proliferation [11].

In addition to aberrant expression of FGF-related genes, mutations of some other genes, e.g., MEN1 and EEF2K, may be associated with the development of PMT and HPT. The multiple endocrine neoplasia type 1 (MEN1) gene encodes menin, a transcription factor expressed in many tissues and involved in the development of endocrine and nonendocrine tumors [12]. Since the cloning of the MEN1 gene, more than 1,500 germline and somatic mutations have been reported, including those found in 35–50% of parathyroid adenomas [12], parathyroid hyperplasia [13], and some mesenchymal tumors [14]. Of relevance, MEN1 mutation has been found in a patient with HPT and TIO due to benign chondroblastoma [13]. The patient has presented with generalized bone pain, difficulties walking, hypophosphatemia, osteomalacia, and high PTH and diagnosed as HPT. He has been treated with a subtotal (three and a half glands) parathyroidectomy and pathology-confirmed hyperplastic lesions in all parathyroid glands [13]. Due to persistent hypophosphatemia, further investigations have been performed and have shown high circulating FGF23 prompting the diagnosis of TIO, while genetic testing has shown somatic mutation of the MEN1 gene in hyperplastic parathyroid lesions. On follow-up, a benign chondroblastoma of the femur has been found and presumed to be the cause of TIO [13].

The EEF2K gene encoding for eukaryotic elongation factor 2 kinase (eEF2K) is suggested to be involved in tumorigenesis [15]. EEF2K is differentially expressed in parathyroid adenomas compared to normal parathyroid tissue and can contribute to the development of parathyroid hyperplasia and HPT [16]. In addition, EEF2K is shown to be important in epithelial-mesenchymal transition (EMT) [15]. The EMT is a highly regulated and reversible process that allows a polarized epithelial cell to undergo multiple biochemical changes resulting in a mesenchymal cell phenotype. The EMT is suggested to play an important role in the development and progression of multiple tumors, including mesenchymal tumors [17]. Of interest, a nonclassical vitamin D pathway is important in promoting epithelial differentiation while diminishing the expression of several EMT inducers and suppressing the EMT process [17]. The available published data can imply that long-standing vitamin D deficiency via its influence on EMT and PTH may contribute to the simultaneous development of PMT and hyperparathyroidism.

In conclusion, the mechanism of PMT tumorigenesis remains elusive, and the hypothesis for the existence of HPT-PMT syndrome remains a hypothesis. Further molecular and experimental studies can improve our understanding and help to discover novel genes and/or pathways to answer the question of whether the association between hyperparathyroidism and PMT is a rarity or a rule.

## Figures and Tables

**Table 1:** Laboratory evaluation.

Measure	Ref	2011^a^	2012^a^	2013^a^	2014^a^	2015^a^	2016^a^	2017^a^	2018^b^	2019^b^	2020^b^
PTH, pg/mL	14–72	114.1	—	110.6	247.6	220.4	—	274.4	—	—	126.3
Ca, mg/dL	8.5–10.1	9.2	8.9	9.2	9.5	9.0	8.3	9.8	10.1	9.9	10.6
PO_4_, mg/dL	2.5–4.9	1.6	1.6	1.0	1.7	0.9	2.3	1.7	3.0	3.0	2.6
Vit D 25(OH)D, ng/mL	30–100	16	—	46	31	32	—	30	30	24	37
Vit D 1,25(OH)_2_, pg/mL	18–72	9	—	27	-	29	—	27	70	37	-
ALP, IU/L	44–174	283	—	252	243	262	—	103	71	71	71
FGF23, RU/mL	0–180	—	—	291	-	-	—	1330	160	174	174
eGFR, mL/min/1.73 m^2^	>60	58	—	85	90	75	66	64	66	66	61
FeP	<20%	—	—	44%	—	—	—	—	—	—	—

ALP, alkaline phosphatase; Ca, calcium; eGFR, estimated glomerular filtration rate; FeP, fractional excretion of phosphate; FGF, fibroblast growth factor; PO_4_, phosphate; PTH, parathyroid hormone; Ref, reference. ^a^Prior to tumor resection. ^b^Following tumor resection.

## Data Availability

No additional data were accessed or generated for this study.
